# Identifying novel Japanese heart failure variants via endothelial *cis*-regulatory element analysis

**DOI:** 10.1093/bioadv/vbag178

**Published:** 2026-06-22

**Authors:** Momoko Hamano, Seitaro Nomura, Kaoru Ito, Ryuichiro Nakato, Issei Komuro, Yoshihiro Yamanishi

**Affiliations:** Department of Bioscience and Bioinformatics, Faculty of Computer Science and Systems Engineering, Kyushu Institute of Technology, Fukuoka 820-8502, Japan; Department of Frontier Cardiovascular Science, The University of Tokyo Graduate School of Medicine, Tokyo 113-8655, Japan; Department of Cardiovascular Medicine, The University of Tokyo Hospital, Tokyo 113-8655, Japan; Laboratory for Cardiovascular Genomics and Informatics, RIKEN Center for Integrative Medical Sciences, Kanagawa 230-0045, Japan; Department of Advanced Biomedical Data Science, Chiba University Graduate School of Medicine, Chiba 260-8670, Japan; Institute for Quantitative Biosciences, The University of Tokyo, Tokyo 113-0032, Japan; Department of Frontier Cardiovascular Science, The University of Tokyo Graduate School of Medicine, Tokyo 113-8655, Japan; International University of Health and Welfare, Tokyo 107-8402, Japan; Department of Bioscience and Bioinformatics, Faculty of Computer Science and Systems Engineering, Kyushu Institute of Technology, Fukuoka 820-8502, Japan; Department of Complex Systems Science, Graduate School of Informatics, Nagoya University, Aichi 464-8601, Japan; Division of Interdisciplinary Research and Development (R&D), Aichi Cancer Center Research Institute, Aichi 464-8681, Japan

## Abstract

**Motivation:**

The detailed molecular mechanisms and disease risk factors for heart failure, especially in the Japanese population, remain to be identified. In this study, we developed a *trans*-omics approach integrating multi-omics data to explore potential disease risk factors on a genome-wide scale. We functionally annotated the single-nucleotide polymorphisms (SNPs) investigated in a Japanese heart failure genome-wide association study using the epigenome data of *cis*-regulatory elements and regulome data of the transcription factor-binding regions identified in vascular endothelial cells.

**Results:**

rs3176334, located in the promoter region of *CDKN1A*, and rs12437763, located in an enhancer, were identified as candidate heart failure-associated SNPs in the Japanese population. Furthermore, the downstream genes regulated by the enhancer containing rs12437763 were predicted to be multiple C2 and *trans*-membrane domain containing two (*MCTP2*) and nuclear receptor subfamily two group F member two (*NR2F2*), both of which are known causative genes for congenital heart disease.

**Availability and implementation:**

These candidate variants are potential risk factors for heart failure in the Japanese population.

## 1 Introduction

Heart failure affects more than 30 million individuals globally, and its prevalence is increasing (Ziaeian and Fonarow 2016). It is speculated that the number of cases of heart failure in Japan will continue to increase with population aging (Shimokawa *et al*. 2015). Despite advancements in treatment, the morbidity and mortality rates of heart failure remain high, with an average 5-year survival rate of 50% (Roger *et al*. 2004). This is attributed to the diverse range of pathological conditions encompassed by heart failure, which is influenced by factors such as hypertension, coronary artery disease, and valvular heart disease. The disease mechanisms of coronary artery disease, the main cause of heart failure, include injury and dysfunction of the vascular endothelium (Higashi and Yoshizumi 2003, Heusch *et al*. 2014). The endothelial cells comprising the vascular endothelium express diverse phenotypes that affect morphology, physiological function, and gene expression patterns in response to extracellular variables such as oxygen concentration, blood pressure, and physiological stress. Thus, it is possible to understand the molecular pathological mechanisms of cardiovascular diseases, including heart failure, in terms of endothelial cells. However, the risk factors for heart failure associated with endothelial cells have not been identified.

There has been a recent focus on genome-wide analyses to identify disease-associated genes, causative loci, and variants. A large-scale genome-wide association study (GWAS) of heart failure in a European population identified 39 risk variants, including 18 novel variants, and seven potential target proteins (CAMK2D, PRKD1, PRKD3, MAPK3, TNFSF12, APOC3, and NAE1; Shah *et al*. 2020, Rasooly *et al*. 2023). A GWAS of heart failure was also conducted in a Japanese cohort, but the only variant that achieved genome-wide significance was the PITX2 region (Ishigaki *et al*. 2020), which corresponds to atrial fibrillation-related genes already identified in the previous European GWAS (Shah *et al*. 2020). These findings confirmed the prevalence of genetic polymorphisms associated with heart failure in European cohorts, but few or no such associations were identified in Japanese cohorts. Furthermore, it must be emphasized that heart failure is a syndrome arising from various cardiac diseases and racial differences in genetic backgrounds. The complexity of heart failure, including diverse etiologies such as myocardial infarction, hinders the identification of genetic statistical observations and significant susceptibility loci through GWAS alone.

The use of GWAS data alone has limited ability to fully elucidate the molecular mechanisms and potential risks of complex diseases. Consequently, the integration of multi-omics data with GWAS is a promising approach to better understand complex diseases (Hasin *et al*. 2017, Akiyama *et al*. 2021). This combination strategy permits the broad exploration of genomic regions such as intergenic and noncoding regions. For example, an integrative analysis of GWAS with *trans*-criptome and epigenome data facilitated the identification of new susceptibility loci for atrial fibrillation (Miyazawa *et al*. 2023). Single-nucleotide polymorphisms (SNPs) located in non-coding regions that do not achieve genome-wide significance thresholds might serve as regulatory SNPs and represent potential disease risks. This indicates that SNPs located in *cis*-regulatory elements (CREs), which are the regions of gene expression regulation, could be candidate regulators of gene expression associated with heart failure. However, heart disease risk SNPs in non-coding regions and their molecular mechanisms remain insufficiently investigated.

In this study, we proposed a *trans*-omics approach to explore the potential risk factors for heart failure by integrating multi-omics data. By focusing on endothelial cells, which are strongly associated with cardiovascular diseases, we functionally annotated SNPs identified in a Japanese heart failure GWAS using the epigenome data of CREs and the regulome data of transcription factor-binding regions detected in endothelial cells. Through this strategy, we identified novel SNPs located in promoter and enhancer regions in endothelial cells as candidate risk factors for heart failure.

## 2 Materials and methods

### 2.1 Acquisition of the GWAS summary

A comprehensive GWAS summary encompassing 22 Japanese diseases was accessed through BioBank Japan (https://biobankjp.org/english/index.html;  Hirata *et al*. 2017, Nagai *et al*. 2017), a research initiative created to investigate the genetic basis of diseases using Japanese genetic resources (Ishigaki *et al*. 2020). For our study, we acquired GWAS summaries related to “heart failure” from a cohort of 2 03 040 healthy individuals and 9413 patients with heart failure.

### 2.2 Visualization of the GWAS summary

We employed the R package “qqman” to visualize the Manhattan and quantile–quantile (Q–Q) plots. Manhattan plots, illustrating linkage disequilibrium (LD) coefficients, genes, and recombination rates near candidate SNPs, were created using LocusZoom (Pruim *et al*. 2011). The Japanese heart failure GWAS summary served as the query, and the LD population was specified as “hg19/1000 Genomes Nov 2014 ASN.”

### 2.3 Acquisition of the epigenomic and *trans*-criptomic data of endothelial cells

Within the framework of the international Human Epigenome Consortium Project (Stunnenberg *et al*. 2016), RNA-seq and ChIP-seq were conducted using human aortic endothelial cells (HAoECs), human coronary artery endothelial cells (HCoAECs), human endocardial cells (HENDCs), human pulmonary artery endothelial cells (HPAECs), human umbilical vein endothelial cells (HUVECs), human umbilical artery endothelial cells (HUAECs), human common carotid artery endothelial cells (HCCaECs), human renal artery endothelial cells (HRAECs), and human great saphenous vein endothelial cells (HGSVECs). The data resulting from these analyses were published in the Gene Expression Omnibus (Nakato *et al*. 2019). For this study, ChIP-seq data for H3K4me3 and H3K27ac were obtained from GSE131953, and RNA-seq data were sourced from the same dataset. A previous study identified 9121 promoter sites and 23 202 enhancer sites specific to endothelial cells through ChIP-seq of H3K4me3 and H3K27ac in nine endothelial cell types (Nakato *et al*. 2019). In the current study, these promoter and enhancer regions of endothelial cells were used to screen for candidate SNPs. The detailed conditions for the analysis of each epigenome and *trans*-criptome dataset were reported previously (Nakato *et al*. 2019).

### 2.4 Acquisition of transcription factor-binding regions in endothelial cells

To identify the transcription factor-binding regions within endothelial cells, we acquired ChIP-seq data annotated with “Coronary artery endothelial cells” from ChIP-Atlas (Oki *et al*. 2018) (http://chip-atlas.org/). The peak caller MACS2 (−log10[Q-value]) was employed through the ChIP-Atlas platform to calculate significance. A threshold of 50 was set as the significance threshold for the transcription factor-binding regions. To define all the transcription factor-binding regions in the endothelial cells, we aggregated the ChIP-seq data annotated with the coronary artery endothelial cells.

### 2.5 Identification of candidate SNPs located in the CRE through functional annotation

Functional annotation was performed to confirm whether the investigated SNPs associated with heart failure were located in the CRE in endothelial cells. In this study, the CRE was defined as the promoter and enhancer regions of endothelial cells. SNPs located within the CRE were extracted and treated as candidate SNPs. The R packages “IRanges (Lawrence *et al*. 2013),” “GenomicRanges (Lawrence *et al*. 2013),” and “GenomicFeatures (Lawrence *et al*. 2013)” were employed for the functional annotation process.

### 2.6 Phenome-wide association study

To assess the potential associations of rs3176334 and rs12437763 with other phenotypes, we investigated phenotype associations using the GWAS atlas (Watanabe *et al*. 2019, Tian *et al*. 2020), and the NHGRI-EBI GWAS catalog dataset served as a reference (Welter *et al*. 2014). The GWAS atlas encompasses 4155 GWAS data for 2960 unique traits, including phenome-wide association study data derived from 1488 electron health records within the Michigan Genomics Initiative (Zhou *et al*. 2018).

### 2.7 Evaluation of eQTLs and sQTLs

To examine the impact of candidate SNPs on gene expression, Genotype Tissue Expression (GTEx) Analysis V7 (https://gtexportal.org/home/;  Lonsdale *et al*. 2013, Aguet *et al*. 2017) was utilized. The statistical significance and effect size of gene expression were determined according to the GTEx project.

### 2.8 Evaluation of the transcription factor binding effects of candidate SNPs

To predict the potential influence of candidate SNPs located in the promoters and enhancers, as presented in Tables 1 and 2, on transcription factor-binding motifs, we calculated the probability using motifbreakR (Coetzee *et al*. 2015). Employing the motifbreakR analysis algorithm, we calculated the extent to which a SNP disrupted or formed new binding motifs for transcription factors. The motifs identified as statistically significant were then visualized as predicted outcomes.

**Table 1:** Candidate SNPs located in endothelial cell promoters.^a^

rsID	Chr.	Position	Alleles REF/ALT	*P*-value	Frequency	Functional gene
rs3176334	6	3 66 48 364	T/C	1.69 × 10^–6^	0.297	Cyclin-dependent kinase inhibitor 1A (CDKN1A)
rs3738067	1	2 90 63 236	G/A	2.94 × 10^–4^	0.796	YTH N6-methyladenosine RNA-binding protein 2 (YTHDF2)
rs35620463	2	21 95 23 439	T/C	3.19 × 10^–4^	0.112	Zinc finger protein 142 (ZNF142)
rs78489660	21	4 52 09 559	C/T	3.51 × 10^–4^	0.144	Ribosomal RNA processing 1 (RRP1)
rs2276244	21	4 52 09 442	C/T	3.54 × 10^–4^	0.144	Ribosomal RNA processing 1 (RRP1)

a List of the top five SNPs with the lowest *P*-value among the SNPs located in the promoter.

**Table 2:** Candidate SNPs located in the endothelial enhancers.^a^

rsID	Chr.	Position	Alleles REF/ALT	*P*-value	Frequency
rs12437763	15	9 51 28 350	A/T	6.48 ×10^–5^	0.201
rs1531732	3	9 90 74 685	A/G	5.41 ×10^–4^	0.235
rs11175272	12	6 45 75 519	C/T	1.06 ×10^–3^	0.072
rs7917309	10	11 41 22 072	C/T	1.16 × 10^–3^	0.204
rs17883795	14	2 09 45 571	G/A	1.19 ×10^–3^	0.082

a List of the top five SNPs with the lowest *P-*value among the SNPs located in the enhancer.

### 2.9 Prediction of chromatin loops and enhancer target genes at candidate SNP locations

To predict chromatin loop formation and the target genes of the enhancer harboring rs12437763, we used CohesinDB (https://cohesindb.iqb.u-tokyo.ac.jp/;  Wang and Nakato 2023). CohesinDB integrates information on cohesin-binding sites and chromatin loops involved in chromatin structure regulation. Using the enhancer (chr15:94582671–94586970) as a query, we predicted the regulatory genes and visualized the identified chromatin loops and associated genes.

### 2.10 Japanese phenome-wide association study of the candidate SNPs

To investigate the potential phenotypic associations of the candidate SNPs in the Japanese population, we used the Japanese GWAS summary statistics available in PheWeb (https://pheweb.jp/). PheWeb offers publicly accessible data encompassing 220 traits specific to the Japanese population. We subjected the candidate SNPs, namely rs3176334 and rs12437763, to a phenome-wide association analysis.

### 2.11 Prediction of miRNA target genes

We employed TargetScanHuman (version 8.0; Lewis *et al*. 2005) to predict the genes targeted by miR-1469. TargetScanHuman evaluates the complementarity of specific sequences in the 3’-terminal untranslated region of mRNA to predict the target genes of various miRNAs. miR-1469 was specified as the query sequence, and the target genes were identified on the basis of a significant interaction (*P* < .05).

### 2.12 Gene Ontology and Kyoto Encyclopedia of Genes and Genome pathway enrichment analysis

To understand the biological functions of the miR-1469 target genes, we used the Database for Annotation, Visualization and Integrated Discovery (https://david.ncifcrf.gov/;  Dennis *et al*. 2003). Gene Ontology (GO) and Kyoto Encyclopedia of Genes and Genome (KEGG) pathway enrichment analyses were performed on the 209 target genes predicted to interact with miR-1469. The functional annotation clustering ranked GO terms and KEGG pathways as statistically significant (*P* < .05), and the results were presented visually using a plot.

## 3 Results

### 3.1 Overview of the proposed method for identifying candidate variants of heart failure in Japanese subjects

**Figure 1:**
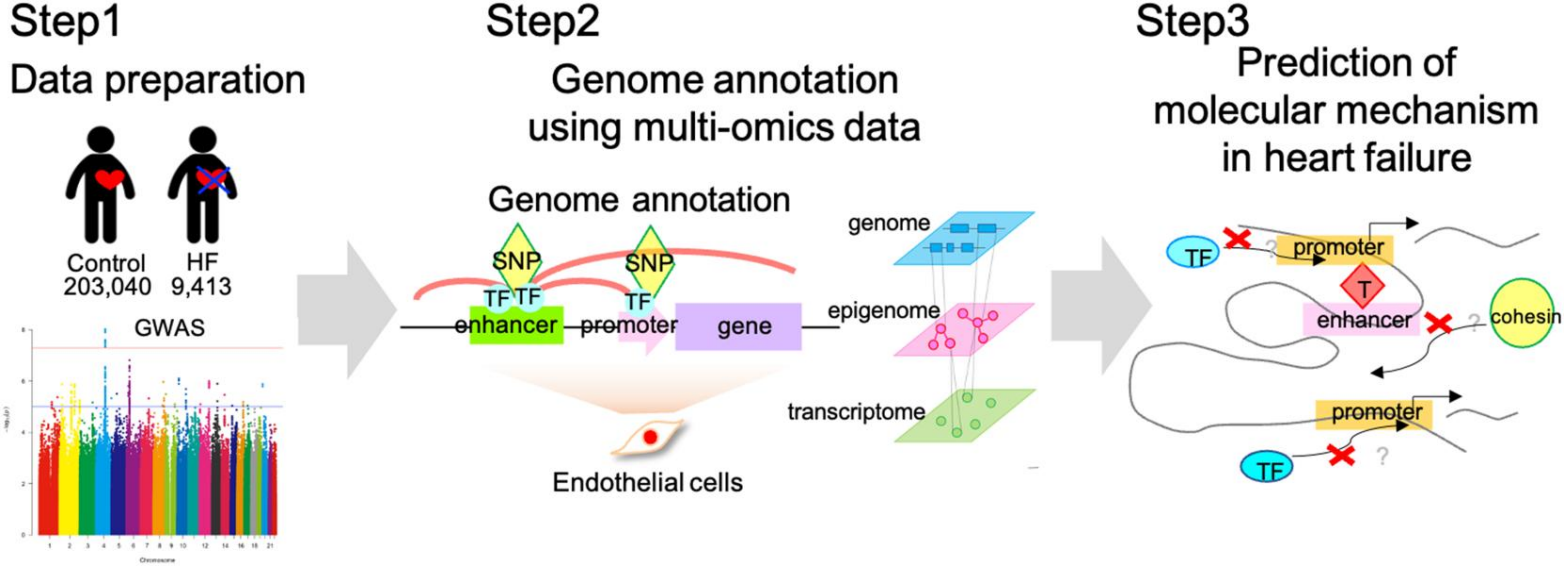
Overview of the proposed methods.

To identify candidate SNPs associated with heart failure in the Japanese population, we focused on vascular endothelial cells because of their crucial role in vascular homeostasis and their association with heart failure (Fig. 1). In the initial step, we explored the susceptibility loci of heart failure using GWAS summaries from the BioBank Japan Project, specifically focusing on Japanese individuals diagnosed with heart failure (Step 1; Ishigaki *et al*. 2020). Subsequently, we identified the CRE region of endothelial cells using epigenome data pertaining to the promoter and enhancer regions of endothelial cell genes (Nakato *et al*. 2019), as well as information on transcription factor-binding regions within these cells (Oki *et al*. 2018). Moreover, we extracted SNPs located in the CRE and the transcription factor-binding regions of endothelial cells (Step 2). In the final step, we predicted the molecular mechanism by which the extracted SNPs could influence the risk of heart failure (Step 3).

In Step 1, we explored the susceptibility loci for heart failure using the GWAS summary for the Japanese population. In Step 2, we identified candidate SNPs associated with heart failure using a *trans*-omics approach integrating epigenome, genome, and *trans*-criptome data. The enhancers, promoters, and transcription factor-binding regions in vascular endothelial cells were examined. In Step 3, we hypothesized the molecular mechanisms by which the identified SNPs could influence the risk of heart failure.

### 3.2 Visualization of disease-associated variants in Japanese patients with heart failure

**Figure 2:**
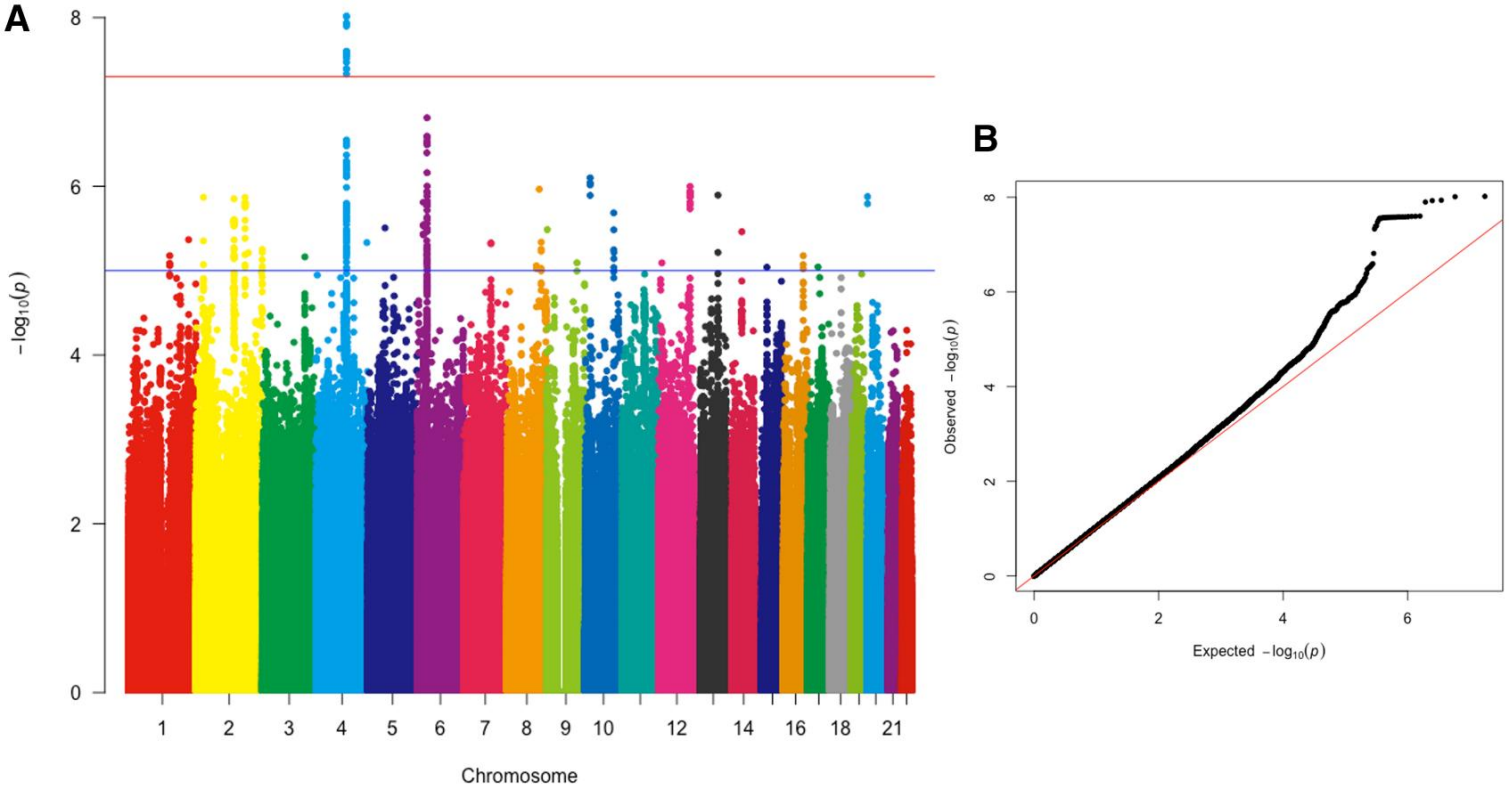
Manhattan and Q–Q plots of the Japanese heart failure GWAS. (A) The Manhattan plot displays the negative logarithm of the *P*-value on the vertical axis, whereas the horizontal axis presents the chromosome number and genomic position. The blue horizontal line denotes *P* = 1.0 × 10^−5^, and the red horizontal line signifies *P* = 1.0 × 10^−8^. (B) The Q–Q plot depicts the distribution of *P*-values in the GWAS. The horizontal axis presents the negative log of the expected *P*-value, and the vertical axis presents the negative logarithm of the observed *P*-value. The red line reflects the null hypothesis of no true associations.

To investigate the SNPs associated with heart failure in the Japanese population, we generated a Manhattan plot using summary statistics derived from a GWAS (Fig. 2A). Notably, SNPs located upstream of *PITX2* on chromosome four were significantly associated with heart failure (Fig. 2A). Subsequently, we employed a Q–Q plot to assess the concordance between the observed *P*-value and the expected distribution (Fig. 2B). The results revealed deviations from the theoretically expected distribution for some data points on the Q–Q plot, indicating an association with heart failure. These findings demonstrate that the GWAS summary comprised variants associated with heart failure.

### 3.3 Extraction of candidate SNPs located in the CRE in endothelial cells

**Figure 3:**
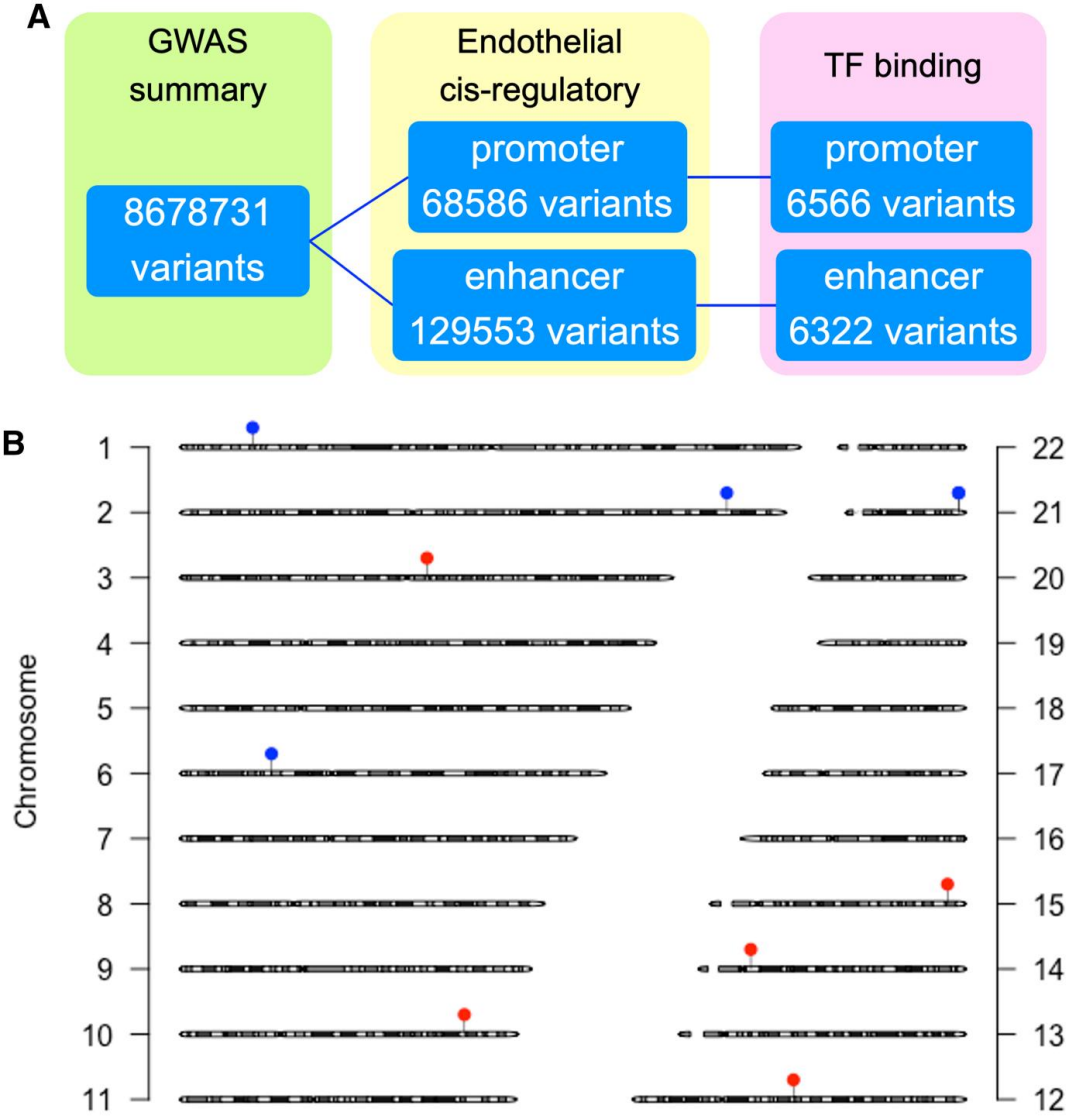
Extraction of candidate SNPs located in the CRE of endothelial cells. Functional annotation of SNPs located in the CRE. (A) The diagram presents the numbers of variants found in the promoter and enhancer and the transcription factor-binding regions. (B) The map of candidate SNPs with low *P*-values on chromosomes among those located in the CRE. Blue pins denote SNPs in the promoter, whereas red pins indicate SNPs in the enhancer.

We annotated 86 78 731 variants analyzed through GWAS based on genomic positions and selectively extracted variants located in the promoter and enhancer regions in endothelial cells along with the transcription factor-binding regions. Specifically, we identified 6566 and 6322 variants in the promoter and enhancer regions, respectively, and both regions encompassed transcription factor-binding regions (Fig. 3A). Tables 1 and 2 outline the top five SNPs with the lowest *P*-value in the promoter and enhancer regions, respectively. The chromosomal locations of these SNPs are depicted in Fig. 3B. Notably, rs3176334, the SNP with the lowest *P-*value in the promoter region, was identified in the *CDKN1A* promoter. The GWAS conducted using a Japanese heart failure population did not reveal susceptibility loci in either the promoter or enhancer regions. In addition, the susceptibility loci identified in the GWAS involving European patients with heart failure (Shah *et al*. 2020, Rasooly *et al*. 2023) were not located within promoters and enhancers featuring transcription factor-binding regions.

### 3.4 Investigation of the phenome-wide associations of candidate heart failure SNPs located in the CRE

We conducted a phenome-wide analysis of rs3176334, which had the lowest *P*-value in the promoter, and rs12437763, which had the lowest *P*-value in the enhancer. This investigation explored the potential associations of these SNPs with other diseases or phenotypes using a comprehensive GWAS summary. rs3176334 displayed a significant association with the QRS interval (*P* = 3.217914 × 10^−9^, Fig. 1A, available as supplementary data at *Bioinformatics Advances* online; Wojcik *et al*. 2019). However, neither rs3176334 nor rs12437763 was associated with heart failure in the phenome-wide analysis (Fig. S2A and B, available as supplementary data at *Bioinformatics Advances* online). Therefore, we extended the phenome-wide analysis to a meta-analysis of the Japanese population, but no variant significantly associated with heart failure emerged. Further investigation illustrated that rs3176334 exhibited stronger associations with chronic heart failure (*P* = 2.00 × 10^−6^) and autoimmune disease (*P* = 2.70 × 10^−6^, Fig. 2A, available as supplementary data at *Bioinformatics Advances* online). Notably, rs3176334 had the strongest associations with cardiac diseases, particularly myocardial infarction (*P* = 7.70 × 10^−4^) and chronic heart failure (*P* = 0.002, Fig. 2B, available as supplementary data at *Bioinformatics Advances* online).

### 3.5 rs3176334, located in the CRE in endothelial cells, qualitatively and quantitatively alters *CDKN1A* expression in the heart and in endothelial cells

To assess the effects of rs3176334 and rs12437763 on gene expression, we utilized GTEx (Lonsdale *et al*. 2013, Aguet *et al*. 2017) to examine expression quantitative trait locus (eQTL) data. rs3176334 exerted eQTL effects across multiple tissues and regulated *CDKN1A* expression (Fig. 1C, available as supplementary data at *Bioinformatics Advances* online). Notably, rs3176334 demonstrated a high *m*-value with a low *P*-value in skeletal muscle (*m* = 0.978) and heart ventricles (*m* = 0.901). Furthermore, we investigated the influence of splicing QTLs (sQTLs) on selective splicing and observed significant effects on splicing patterns in the tibial artery (Fig. 1D, available as supplementary data at *Bioinformatics Advances* online), aorta (Fig. 1E, available as supplementary data at *Bioinformatics Advances* online), and heart (Fig. 1F, available as supplementary data at *Bioinformatics Advances* online), influencing the overall splicing pattern. As *CDKN1A* expression in endothelial cells remained consistent across all cell types (Fig. 3A, available as supplementary data at *Bioinformatics Advances* online), our findings suggest that rs3176334 both quantitatively and qualitatively altered the gene function of *CDKN1A* in endothelial cells.

### 3.6 SNPs located in the CRE in endothelial cells exhibit high LD coefficients, and they are located in a region of epigenomic variation

**Figure 4:**
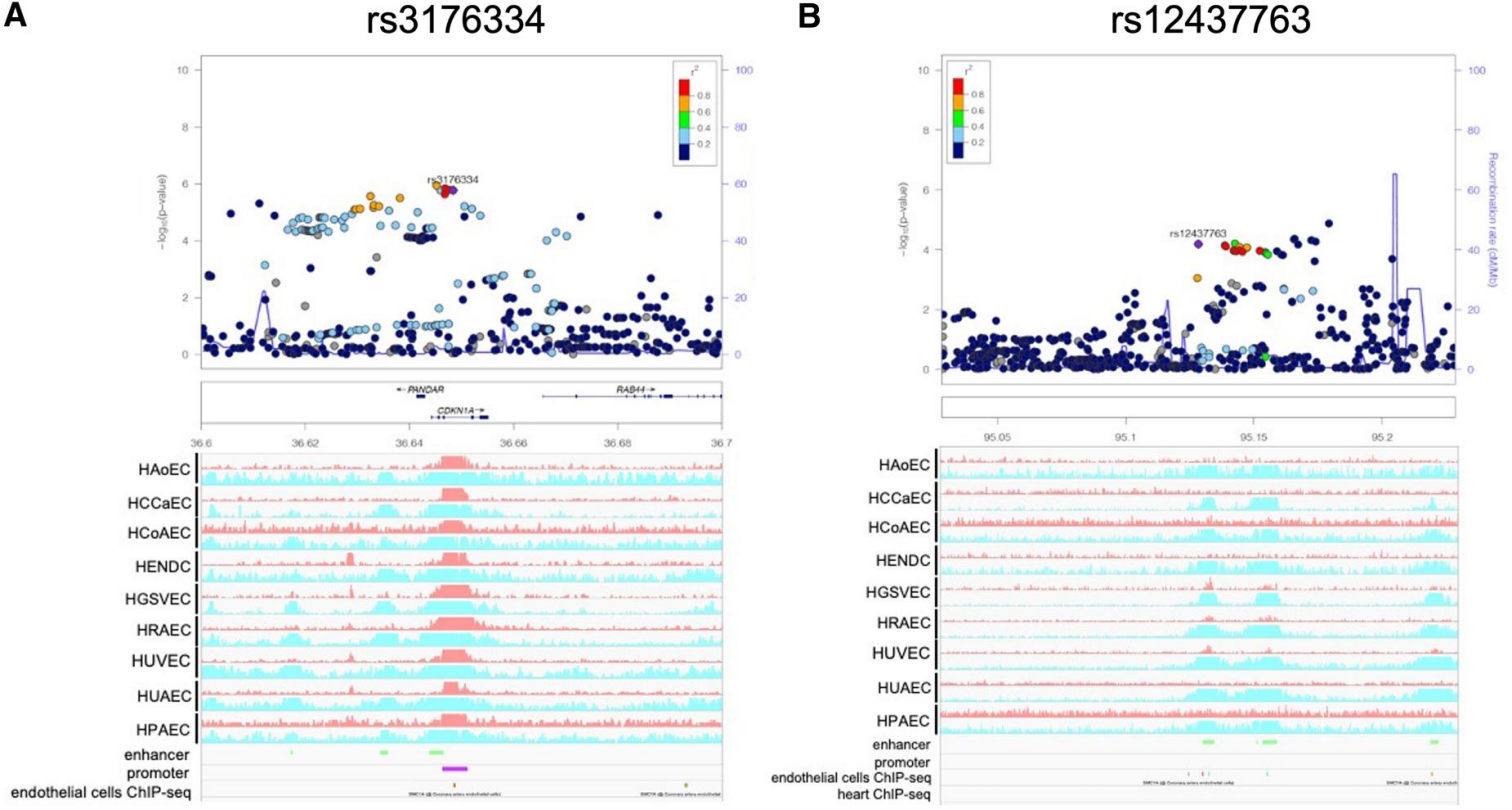
Associations of candidate SNPs with histone modification in the genome. (A) The Manhattan plot in proximity to rs3176334 includes visualizations of the LD coefficient, epigenetic modifications (H3K4me3 and H3K27ac) in nine endothelial cell types, and transcription factor-binding regions. The color of the dots in the upper Manhattan plot signifies the strength of the LD coefficient. Enhancer and promoter regions in the endothelial cells are denoted by green and purple bars, respectively, and the ChIP-seq rows represent the transcription factor-binding regions in the endothelial cells. (B) The Manhattan plot near rs12437763 displays the LD coefficient, epigenetic modifications (H3K4me3 and H3K27ac) in endothelial cells, and transcription factor-binding regions. ChIP-seq rows indicate transcription factor-binding regions in both endothelial cells and the heart.

We investigated the LD coefficients and epigenome patterns near the rs3176334 and rs12437763 loci (Fig. 4). Both rs3176334 and rs12437763 displayed high LD coefficients (*r*^2^ = 1.0). We examined the epigenome states (H3K4me3 and H3K27ac) at the rs3176334 and rs12437763 loci in endothelial cells derived from various tissue sources [HAoECs, HCoAECs, HENDCs, HPAECs, HUVECs, HUAECs, HCCaECs, HRAECs, and HGSVECs]. The results indicated that the prominent H3K4me3 and H3K27ac peaks in all endothelial cells were near rs3176334 (Fig. 4A). Regarding rs12437763, the enhancer was identified as a transcription factor-binding region in endothelial cells but not in the heart (Fig. 4B). Thus, rs12437763 is a SNP located in an endothelial cell-specific gene regulatory region. In summary, our findings indicated that the enhancer at rs12437763 serves as an endothelial cell-specific gene regulatory region.

### 3.7 SNPs located in the CRE in endothelial cells affect transcription factor binding

**Figure 5:**
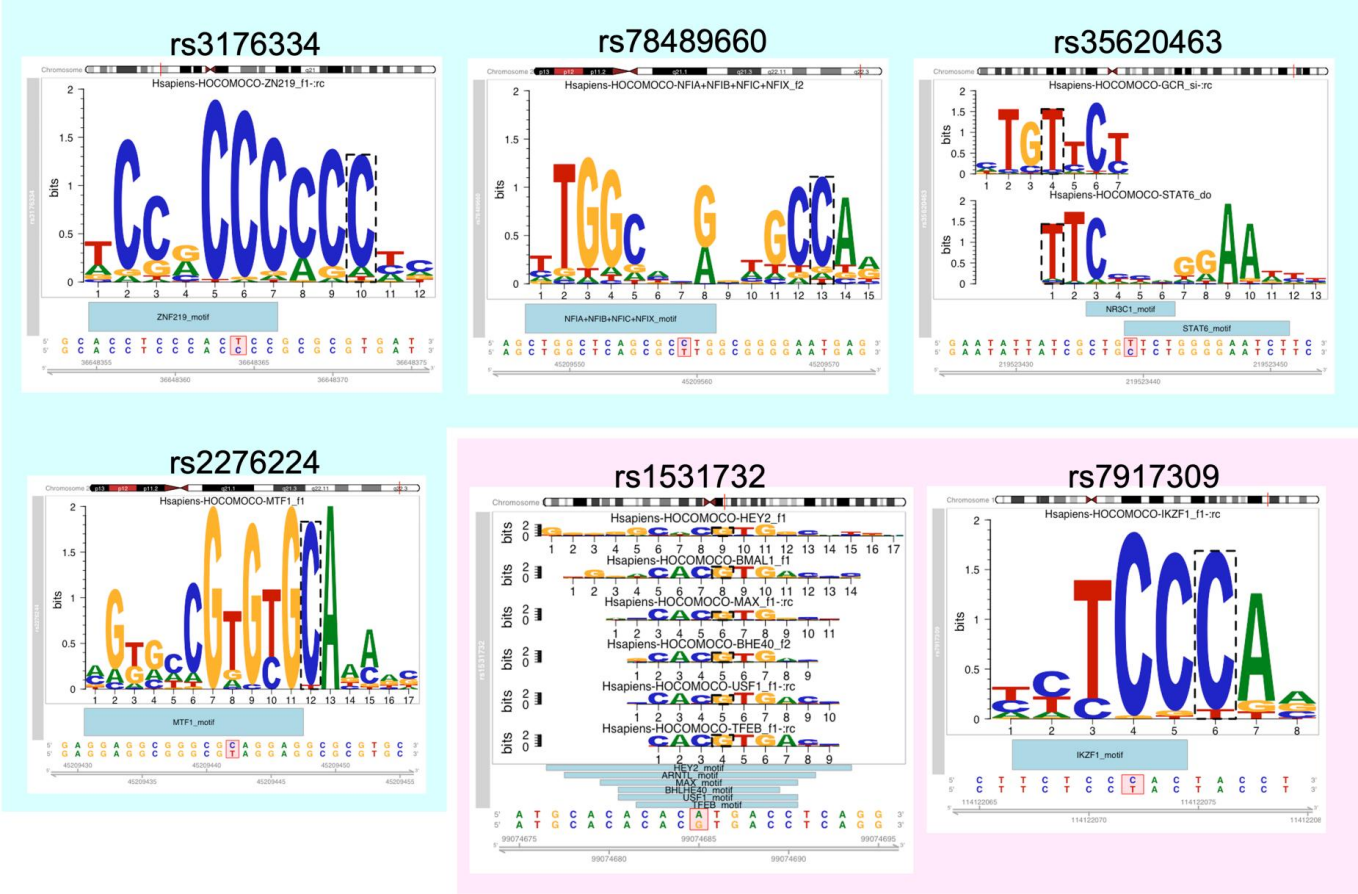
Transcription factor-binding motifs located near the candidate SNPs. The diagram displays the genomic coordinates of nearby SNPs within the CRE predicted to influence transcription factor motif binding, namely rs3176334, rs78489660, rs35620463, rs2276224, rs1531732, and rs7917309. The positions of these SNPs in the motifs are delineated by dotted boxes in the motif logos and red boxes in the genome sequences, with reference and alternative alleles denoted at the bottom. The genomic sequence and coordinates are presented at the bottom of each display, and the aqua boxes indicate the positions at which the motifs match. The locations of rs3176334, rs78489660, rs35620463, and rs2276224 in the promoters are highlighted in light blue, whereas the locations of rs1531732 and rs7917309 in the enhancers are highlighted in pink.

We investigated whether the candidate SNPs could disrupt transcription factor binding using motifbreakR. Specifically, rs3176334, rs78489660, rs35620463, and rs2276224 in the promoter and rs1531732 and rs7917309 in the enhancer had strong effects on transcription factor binding (Fig. 5). Notably, rs3176334 had a strong effect on the probability of ZNF219 binding. ZNF219 acts as a transcriptional repressor, implying that rs3176334 can regulate *CDKN1A* transcription by affecting the binding capability of ZNF219. In addition, candidate SNPs located in enhancers, such as rs1531732, were identified in regions with multiple transcription factor binding sites, indicating that these candidate SNPs influence gene expression.

### 3.8 The enhancer in which rs12437763 is located forms a chromatin loop and regulates *MCTP2* and *NR2F2* expression

In the transcription factor binding analyses, rs12437763 did not affect the affinity of the transcription factors binding near rs12437763. Thus, the potential of rs12437763 as a risk factor for heart failure cannot be explained by transcription factor binding. Therefore, we focused on the enhancer in which rs12437763 is located. We hypothesized that the pathological mechanism by which rs12437763 acts as a risk factor might be explained through gene expression alterations regulated by this enhancer. We investigated the genes regulated by the enhancer using CohesinDB (Wang and Nakato 2023).

**Figure 6:**
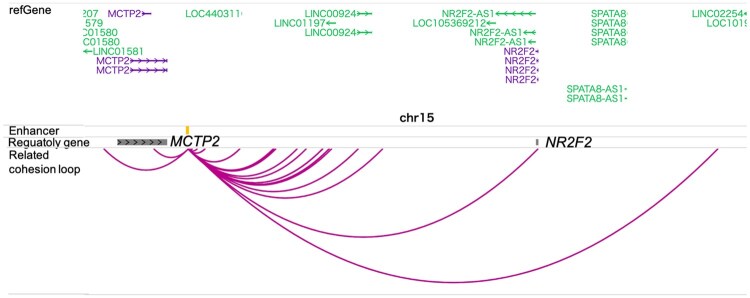
Prediction of genes regulated by enhancers in which rs12437763 is located. The upper panel displays the positions of the reference genes. Genes predicted to be regulated by rs12437763 are denoted in purple, other nearby genes are presented in green, and the regulatory genes are represented by gray bars. The bottom panel illustrates chromatin loops predicted to interact with the enhancer, as depicted by purple lines.

The predictive results revealed that the enhancer containing rs12437763 forms multiple chromatin loops and regulates genes linked to congenital heart disease, including nuclear receptor subfamily 2 group F member 2 (*NR2F2*) and multiple C2 and *trans*-membrane domain containing 2 (*MCTP2*; Fig. 6). The alteration in *NR2F2* expression was presumed to be associated with the expression of miR-1469, which is generated from an *NR2F2* intron. To link altered miR-1469 expression to heart failure, we predicted the regulatory genes targeted by miR-1469 using TargetScanHuman (Agarwal *et al*. 2015). We identified 209 genes that interacted with miR-1469 (Fig. 4, available as supplementary data at *Bioinformatics Advances* online). GO and KEGG pathway enrichment analyses of these genes indicated their involvement in several GO pathways, including “regulation of transcription from the RNA polymerase II promoter” (Fig. 5A, available as supplementary data at *Bioinformatics Advances* online). Additionally, GO terms related to cardiac and muscle protein organization, such as “heart development” and “actin cytoskeleton organization,” were identified. The most significantly enriched KEGG pathway was “endocytosis” (Fig. 5B, available as supplementary data at *Bioinformatics Advances* online). These results suggested that rs12437763 serves as a potential risk factor for heart failure by influencing the expression of genes associated with heart disease, such as *NR2F2* and *MCTP2*, and by modulating multiple biological functions through miR-1469.

## 4 Discussion

In this study, we developed a novel in silico *trans*-omics approach that integrated multi-omics data of heart failure, a condition for which significant susceptibility loci are difficult to identify by GWAS alone. By focusing on endothelial cells, which are strongly associated with the pathogenesis of cardiovascular diseases, we successfully identified potential novel genetic polymorphisms associated with heart failure. The candidate SNPs identified in promoter and enhancer regions in endothelial cells in this study were not detected by exploring GWAS data alone. Furthermore, the candidate SNP in the promoter was located near a known heart failure susceptibility locus, whereas the candidate SNP in the enhancer is postulated to regulate genes known to cause heart disease. These variants could be potential risk candidates for heart failure.

Endothelial cells have been found to play a critical role in the pathogenesis of heart failure. Progressive endothelial dysfunction affects the cardiovascular system (Marti *et al*. 2012, Heusch *et al*. 2014). However, the association between genetic polymorphisms derived from endothelial cells and heart failure remains unclear. The molecular pathological mechanisms triggered by these polymorphisms are also poorly understood. In this study, we identified SNPs located in promoters and enhancers in endothelial cells from the heart failure GWAS summary and elucidated the molecular mechanisms underlying the disease risks utilizing a *trans*-omics approach. Our findings both identified new risk factors for heart failure and revealed novel molecular mechanisms of heart failure with a focus on vascular endothelial cells.

rs3176334, identified within the *CDKN1A* promoter region of endothelial cells, aligns with a heart failure-related gene revealed in a European heart failure meta-analysis (rs4135240; chr6: 3 66 47 680 *P* = 6.84 × 10^−9^; Shah *et al*. 2020). *CDKN1A*, a cell cycle inhibitor, induces cell cycle arrest in cardiomyocytes after birth (Tane *et al*. 2014). *CDKN1A* expression is triggered by DNA damage during cardiac hypertrophy through the activation of the transcription factor p53 (Nomura *et al*. 2018). miR-208b, which regulates *CDKN1A*, influences endothelial cell proliferation by disrupting *CDKN1A* expression (Jiang *et al*. 2021), and it is associated with myocardial infarction and cardiac hypertrophy (Zhou *et al*. 2017, Wang *et al*. 2019). Thus, miR-208b could be a risk factor for Japanese heart failure through its modulation of *CDKN1A* expression. Notably, rs3176334 did not emerge as a risk factor in the previous European GWAS (Shah *et al*. 2020).

The enhancer harboring rs12437763 was predicted to regulate *NR2F2* and *MCTP2*. Copy number variation and mutations in *MCTP2* are known causes of congenital heart disease, such as mitral regurgitation and stenosis, and *MCTP2* knockdown in *Xenopus laevis* embryos led to endocardial defects (Lalani *et al*. 2013). *NR2F2* (COUP-TFII) was additionally identified as a causative gene in congenital heart disease (Al Turki *et al*. 2014). Rare variants of the nuclear receptor *NR2F2* can result in human congenital heart disease through the altered expression of various genes (Al Turki *et al*. 2014). *MCTP2* and *NR2F2* expression varied across different cell types, being low in HUAECs but high in specific cell types derived from the upper body (Fig. 3B and C, available as supplementary data at *Bioinformatics Advances* online). *NR2F2* binds to vein-specific endothelial cell enhancers, promoting vein-related gene expression (Sissaoui *et al*. 2020), highlighting its involvement in regulating vein-related gene expression. These observations suggest that the enhancer containing rs12437763 is a potential risk factor for cardiac disease through its ability to alter cardiac disease-associated gene expression. Conversely, miR-1469, derived from an *NR2F2* intron, has not been directly linked to cardiac diseases. miR-1469 represses Mcl1 expression and induces apoptosis in pharyngeal carcinoma cells in response to p53 (Ma *et al*. 2018). Our study revealed that the target genes of miR-1469 regulate several genes involved in heart development. Furthermore, endocytosis emerged as a KEGG pathway enriched in genes targeted by miR-1469, a process associated with cardiovascular disease through the dysfunction of several endocytic proteins, including sorting nexin (SNX), which triggers inflammatory responses in endothelial cells (Yang *et al*. 2019). SNX1 was predicted as a target of miR-1469 (Fig. 4, available as supplementary data at *Bioinformatics Advances* online). We hypothesize that alterations in *NR2F2* expression levels affect cardiovascular function through altering NR2F2 protein function and influencing miR-1469 generation. These findings demonstrated that rs12437763 is a potential risk factor for heart failure in the Japanese population (Fig. 6, available as supplementary data at *Bioinformatics Advances* online).

The limitation of the proposed method in this study is its dependence on data availability. In this *trans*-omics approach, *trans*-criptome, and epigenome data were generated using the endothelial cells of healthy individuals. Because the epigenomic status of endothelial cells can change under heart failure, the acquisition of multi-omics data from the endothelial cells of patients with heart failure could enable the elucidation of disease-relevant molecular mechanisms and the identification of novel pathogenic candidates. Additionally, to identify susceptibility loci for heart failure, which features diverse pathologies, in the Japanese population, it will be necessary to investigate GWAS summaries with further stratification of conditions such as myocardial infarction and atrial fibrillation. Finally, the *trans*-omics approach developed in this study can be applied to other diseases. By identifying potential risk candidates that cannot be detected with single-omics data, the proposed method is expected to contribute to precision medicine in terms of disease onset, early diagnosis, and prognosis prediction in the future.

## Supplementary Material

vbag178_Supplementary_Data

## Data Availability

No publicly available data were generated in this study.

## References

[vbag178-B1] Agarwal V , Bell GW, Nam JW et al Predicting effective microRNA target sites in mammalian mRNAs. Elife 2015;4:e05005. 26267216 10.7554/eLife.05005 PMC4532895

[vbag178-B2] Aguet F , Brown AA, Castel SE et al Genetic effects on gene expression across human tissues. Nature 2017;550:204–13. 29022597 10.1038/nature24277 PMC5776756

[vbag178-B3] Akiyama M. Multi-omics study for interpretation of genome-wide association study. J Hum Genet. 2021;66:3–10. 32948838 10.1038/s10038-020-00842-5

[vbag178-B4] Al Turki S , Manickaraj AK, Mercer CL et al Rare variants in NR2F2 cause congenital heart defects in humans. Am J Hum Genet. 2014;94:574–85. 24702954 10.1016/j.ajhg.2014.03.007 PMC3980509

[vbag178-B5] Coetzee SG , Coetzee GA, Hazelett DJ. MotifbreakR: an R/bioconductor package for predicting variant effects at transcription factor binding sites. Bioinformatics 2015;31:3847–9. 26272984 10.1093/bioinformatics/btv470 PMC4653394

[vbag178-B6] Dennis G , ShermanBT, HosackDA et al DAVID: database for annotation, visualization, and integrated discovery. Genome Biol 2003;4:1–11.12734009

[vbag178-B7] Hasin Y , Seldin M, Lusis A. Multi-omics approaches to disease. Genome Biol 2017;18:83–15. 28476144 10.1186/s13059-017-1215-1 PMC5418815

[vbag178-B8] Heusch G , Libby P, Gersh B et al Cardiovascular remodelling in coronary artery disease and heart failure. Lancet 2014;383:1933–43. 24831770 10.1016/S0140-6736(14)60107-0 PMC4330973

[vbag178-B9] Higashi Y , Yoshizumi M. Endothelial function: from vascular biology to clinical applications. Nippon Rinsho 2003;61:1138–44. 12877075

[vbag178-B10] Hirata M , Kamatani Y, Nagai A et al Cross-sectional analysis of BioBank Japan clinical data: a large cohort of 200,000 patients with 47 common diseases. J Epidemiol 2017;27:S9, S21. 28190657 10.1016/j.je.2016.12.003 PMC5363792

[vbag178-B12] Ishigaki K , Akiyama M, Kanai M et al Large-scale genome-wide association study in a Japanese population identifies novel susceptibility loci across different diseases. Nat Genet 2020;52:669–79. 32514122 10.1038/s41588-020-0640-3 PMC7968075

[vbag178-B13] Jiang W , Song Q, Lu Z et al Myocardial infarction-associated extracellular vesicle-delivered miR-208b affects the growth of human umbilical vein endothelial cells via regulating CDKN1A. Biomed Res Int 2021;2021:9965639. 34195287 10.1155/2021/9965639 PMC8203352

[vbag178-B14] Lalani SR , Ware SM, Wang X et al MCTP2 is a dosage-sensitive gene required for cardiac outflow tract development. Hum Mol Genet 2013;22:4339–48. 23773997 10.1093/hmg/ddt283 PMC3792692

[vbag178-B15] Lawrence M , Huber W, Pages H et al Software for computing and annotating genomic ranges. PLoS Comput Biol 2013;9:e1003118. 23950696 10.1371/journal.pcbi.1003118 PMC3738458

[vbag178-B16] Lewis BP , Burge CB, Bartel DP. Conserved seed pairing, often flanked by adenosines, indicates that thousands of human genes are microRNA targets. Cell 2005;120:15–20. 15652477 10.1016/j.cell.2004.12.035

[vbag178-B17] Lonsdale J , Thomas J, Salvatore M et al The Genotype-Tissue expression (GTEx) project. Nat Genet 2013;45:580–5. 23715323 10.1038/ng.2653 PMC4010069

[vbag178-B18] Ma C , Zhang Y, Tang L et al MicroRNA-1469, a p53-responsive microRNA promotes Genistein induced apoptosis by targeting Mcl1 in human laryngeal cancer cells. Biomed Pharmacother 2018;106:665–71. 29990856 10.1016/j.biopha.2018.07.005

[vbag178-B19] Marti CN , Gheorghiade M, Kalogeropoulos AP et al Endothelial dysfunction, arterial stiffness, and heart failure. J Am Coll Cardiol 2012;60:1455–69. 22999723 10.1016/j.jacc.2011.11.082

[vbag178-B20] Miyazawa K , Ito K, Ito M et al Cross-ancestry genome-wide analysis of atrial fibrillation unveils disease biology and enables cardioembolic risk prediction. Nat Genet 2023;55:187–97. 36653681 10.1038/s41588-022-01284-9 PMC9925380

[vbag178-B21] Nagai A , Hirata M, Kamatani Y et al Overview of the BioBank Japan project: study design and profile. J Epidemiol. 2017;27:S2, S8. 28189464 10.1016/j.je.2016.12.005 PMC5350590

[vbag178-B22] Nakato R , Wada Y, Nakaki R et al Comprehensive epigenome characterization reveals diverse transcriptional regulation across human vascular endothelial cells. Epigenetics Chromatin 2019;12:77–16. 31856914 10.1186/s13072-019-0319-0 PMC6921469

[vbag178-B23] Nomura S , Satoh M, Fujita T et al Cardiomyocyte gene programs encoding morphological and functional signatures in cardiac hypertrophy and failure. Nat Commun 2018;9:4435–17. 30375404 10.1038/s41467-018-06639-7 PMC6207673

[vbag178-B24] Oki S , Ohta T, Shioi G et al ChIP-Atlas: a data-mining suite powered by full integration of public ChIP-seq data. EMBO Rep 2018;19:1–10. 30413482 10.15252/embr.201846255 PMC6280645

[vbag178-B25] Pruim RJ , Welch RP, Sanna S et al LocusZoom: regional visualization of genome-wide association scan results. Bioinformatics 2011;27:2336–7. 10.1093/bioinformatics/btq419 PMC2935401 20634204

[vbag178-B26] Rasooly D , Peloso GM, Pereira AC et al Genome-wide association analysis and Mendelian randomization proteomics identify drug targets for heart failure. Nat Commun 2023;14:3826. 37429843 10.1038/s41467-023-39253-3 PMC10333277

[vbag178-B27] Roger VL , Weston SA, Redfield MM et al Trends in heart failure incidence and survival in a community-based population. JAMA 2004;292:344–50. 15265849 10.1001/jama.292.3.344

[vbag178-B28] Shah S , Henry A, Roselli C et al Genome-wide association and Mendelian randomisation analysis provide insights into the pathogenesis of heart failure. Nat Commun 2020;11:163–12. 31919418 10.1038/s41467-019-13690-5 PMC6952380

[vbag178-B29] Shimokawa H , Miura M, Nochioka K et al Heart failure as a general pandemic in Asia. Eur J Heart Fail 2015;17:884–92. 26222508 10.1002/ejhf.319

[vbag178-B30] Sissaoui S , Yu J, Yan A et al Genomic characterization of endothelial enhancers reveals a multifunctional role for NR2F2 in regulation of arteriovenous gene expression. Circ Res 2020;126:875–88. 32065070 10.1161/CIRCRESAHA.119.316075 PMC7212523

[vbag178-B31] Stunnenberg HG , Abrignani S, Adams D et al The International Human Epigenome Consortium: a blueprint for scientific collaboration and discovery. Cell 2016;167:1145–9. 27863232 10.1016/j.cell.2016.11.007

[vbag178-B32] Tane S , Ikenishi A, Okayama H et al CDK inhibitors, p21Cip1 and p27Kip1, participate in cell cycle exit of mammalian cardiomyocytes. Biochem Biophys Res Commun 2014;443:1105–9. 24380855 10.1016/j.bbrc.2013.12.109

[vbag178-B33] Tian D , Wang P, Tang B et al GWAS Atlas: a curated resource of genome-wide variant-trait associations in plants and animals. Nucleic Acids Res. 2020;48:D927–D932. 31566222 10.1093/nar/gkz828 PMC6943065

[vbag178-B34] Wang J , Nakato R. CohesinDB: a comprehensive database for decoding cohesin-related epigenomes, 3D genomes and transcriptomes in human cells. Nucleic Acids Res 2023;51:D70–D79. 36162821 10.1093/nar/gkac795 PMC9825609

[vbag178-B35] Wang J , Song C, Cao X et al MiR-208b regulates cell cycle and promotes skeletal muscle cell proliferation by targeting CDKN1A. J Cell Physiol 2019;234:3720–9. 30317561 10.1002/jcp.27146

[vbag178-B36] Watanabe K , Stringer S, Frei O et al A global overview of pleiotropy and genetic architecture in complex traits. Nat Genet 2019;51:1339–48. 31427789 10.1038/s41588-019-0481-0

[vbag178-B37] Welter D , MacArthur J, Morales J et al The NHGRI GWAS catalog, a curated resource of SNP-trait associations. Nucleic Acids Res 2014;42:D1001–1006. 24316577 10.1093/nar/gkt1229 PMC3965119

[vbag178-B38] Wojcik GL , Graff M, Nishimura KK et al Genetic analyses of diverse populations improves discovery for complex traits. Nature 2019;570:514–8. 31217584 10.1038/s41586-019-1310-4 PMC6785182

[vbag178-B39] Yang J , Anthony V, Villar M et al The emerging role of sorting nexins in cardiovascular diseases. Clin Sci 2019;133:723–37. 10.1042/CS20190034 PMC6418407 30877150

[vbag178-B40] Zhou W , Nielsen JB, Fritsche LG et al Efficiently controlling for case-control imbalance and sample relatedness in large-scale genetic association studies. Nat Genet 2018;50:1335–41. 30104761 10.1038/s41588-018-0184-y PMC6119127

[vbag178-B41] Zhou Q , Schötterl S, Backes D et al Inhibition of miR-208b improves cardiac function in titin-based dilated cardiomyopathy. Int J Cardiol 2017;230:634–41. 28065693 10.1016/j.ijcard.2016.12.171

[vbag178-B42] Ziaeian B , Fonarow GC. Epidemiology and aetiology of heart failure. Nat Rev Cardiol 2016;13:368–78. 26935038 10.1038/nrcardio.2016.25 PMC4868779

