# Supplementary material for: Identifying novel Japanese heart failure variants via endothelial cis-regulatory element analysis
Source: Bioinform Adv. 2026 Jun 22;6(1):vbag178. doi: 10.1093/bioadv/vbag178 (PMC13348845; doi:10.1093/bioadv/vbag178)
Supplement: vbag178_Supplementary_Data [file vbag178_supplementary_data.zip › 30-Jun-2026_125102_supplementary_figures_proof2.docx]

**Supplementary Figure**

**Identifying novel Japanese heart failure variants via endothelial cis-regulatory element analysis**

Momoko Hamano^1*^, Seitaro Nomura^2,3^, Kaoru Ito^4,5^, Ryuichiro Nakato^6^, Issei Komuro^2,7^, and Yoshihiro Yamanishi^1,8,9*^

^1^Department of Bioscience and Bioinformatics, Faculty of Computer Science and Systems Engineering, Kyushu Institute of Technology, Fukuoka 820-8502, Japan

^2^Department of Frontier Cardiovascular Science, The University of Tokyo Graduate School of Medicine, Tokyo, 113-8655, Japan

^3^Department of Cardiovascular Medicine, The University of Tokyo Hospital, Tokyo, 113-8655, Japan

^4^Laboratory for Cardiovascular Genomics and Informatics, RIKEN Center for Integrative Medical Sciences, Kanagawa 230-0045, Japan

^5^Department of Advanced Biomedical Data Science, Chiba University Graduate School of Medicine, Chiba, 260-8670, Japan

^6^Institute for Quantitative Biosciences, The University of Tokyo, Tokyo, 1-1-1, Tokyo 113-0032, Japan

^7^International University of Health and Welfare, 107-8402 Tokyo, Japan

^8^Department of Complex Systems Science, Graduate School of Informatics, Nagoya University, Aichi 464-8601, Japan

^9^ Division of Interdisciplinary Research and Development (R&D), Aichi Cancer Center Research Institute, Aichi 464-8681, Japan

rs3176334

A


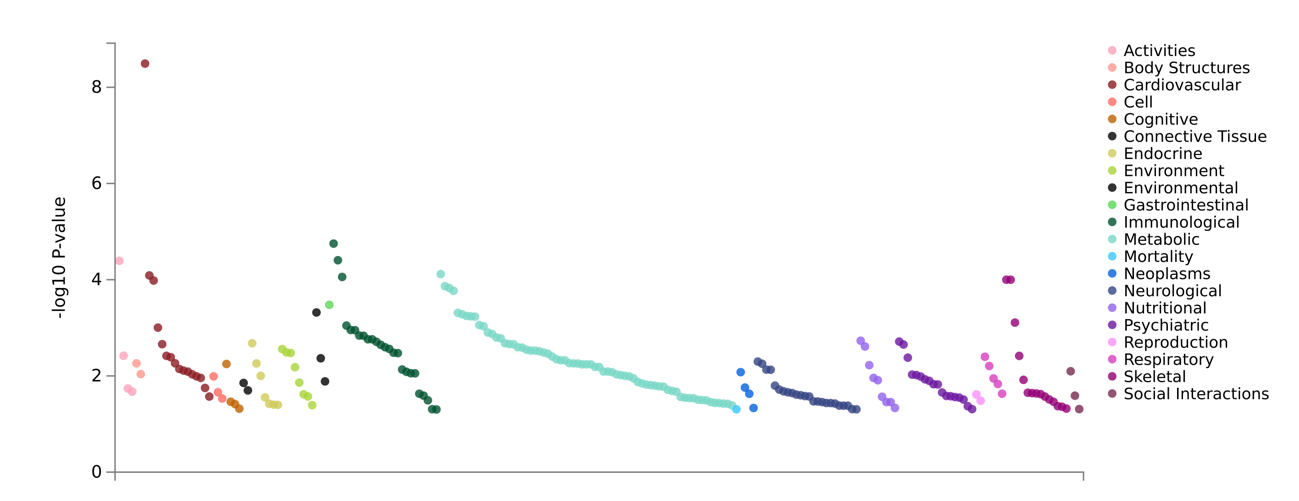

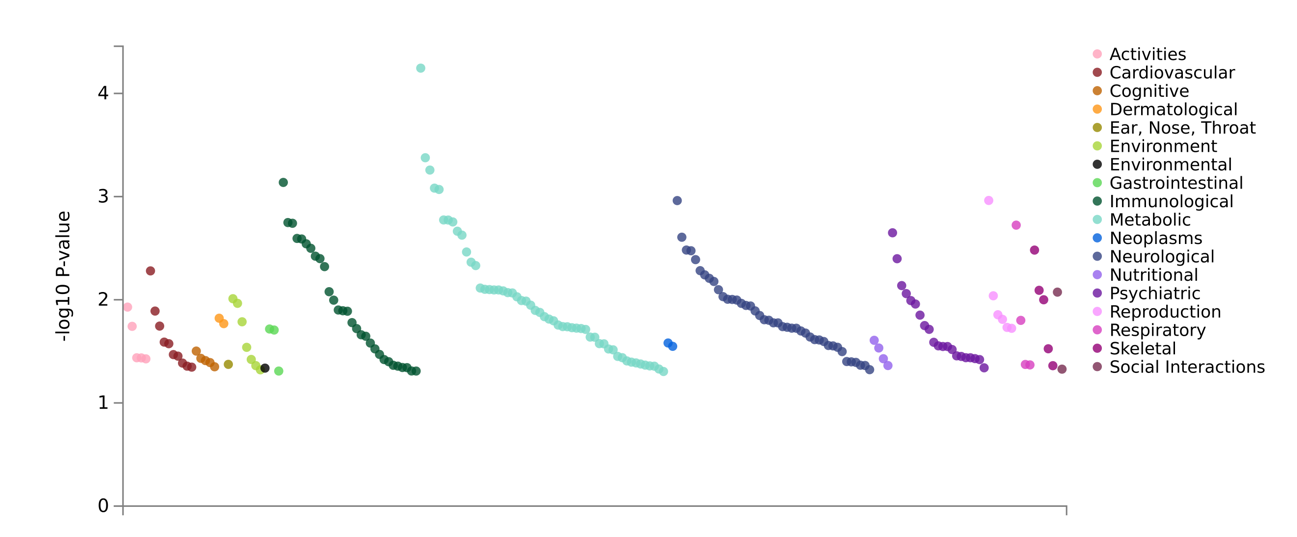


B

rs12437763

QRS interval


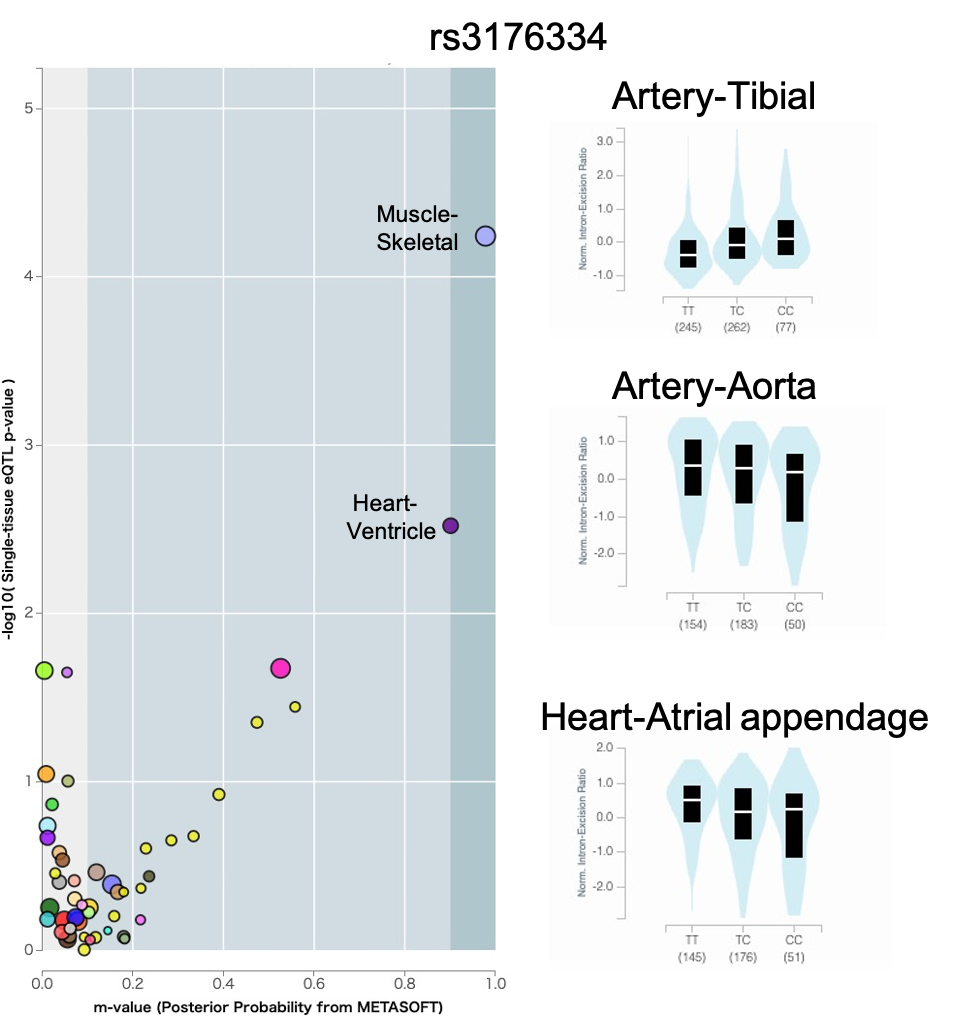


D

C

*p*=2.07e-8

*p=1*.3e-12

F

E

*p=1.*91e-10

**Supplementary Figure 1 Functional evaluation of the effects of candidate SNPs on phenotype and gene expression**

(A) Phenome-wide association study of rs3176334 using the GWAS atlas. The vertical axis presents the negative logarithm of the *p*-value, and the color of the dots indicates the phenotype categories. (B) Phenome-wide association analysis of rs12437763. (C) Multi-tissue eQTL effects of rs3176334 on *CDKN1A*. The vertical axis depicts the negative logarithm of the *p*-value, and the horizontal axis presents the *m*-value (posterior probability of an eQTL effect in each tissue confirmed in the cross-tissue meta-analysis). Each dot corresponds to an individual tissue. (D) Violin plot of *CDKN1A* for the sQTL rs3176334 in the tibial artery. The vertical axis denotes the normalized intron-to-exon ratio, and the horizontal axis depicts the genotype. All statistical data originated from the GTEx project (version 7). (E) Violin plot of *CDKN1A* for the sQTL rs3176334 in the aorta. (F) Violin plot of *CDKN1A* for the sQTL rs3176334 in the atrial appendage.


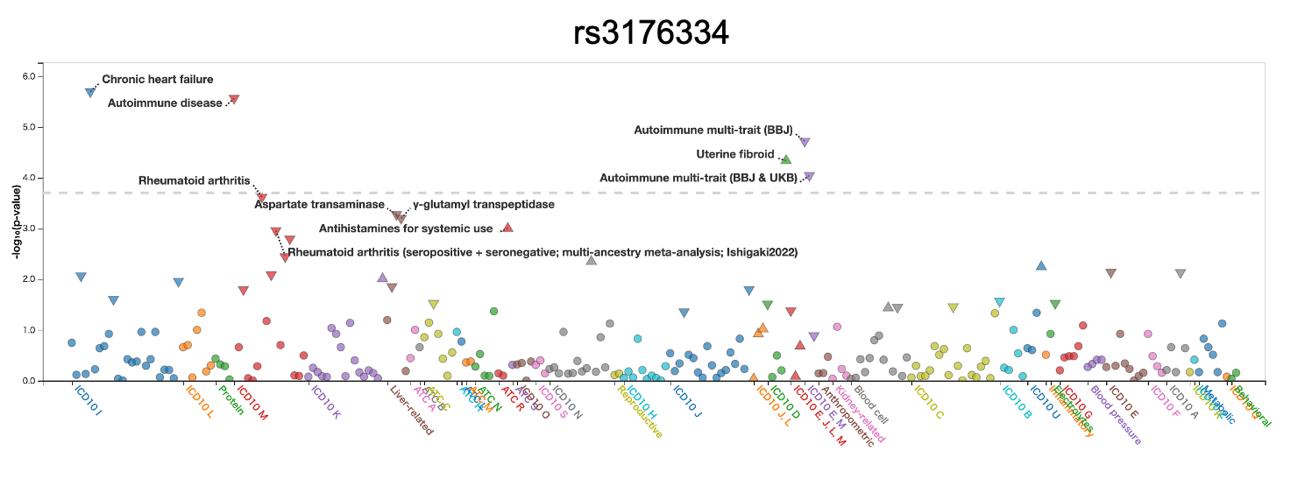

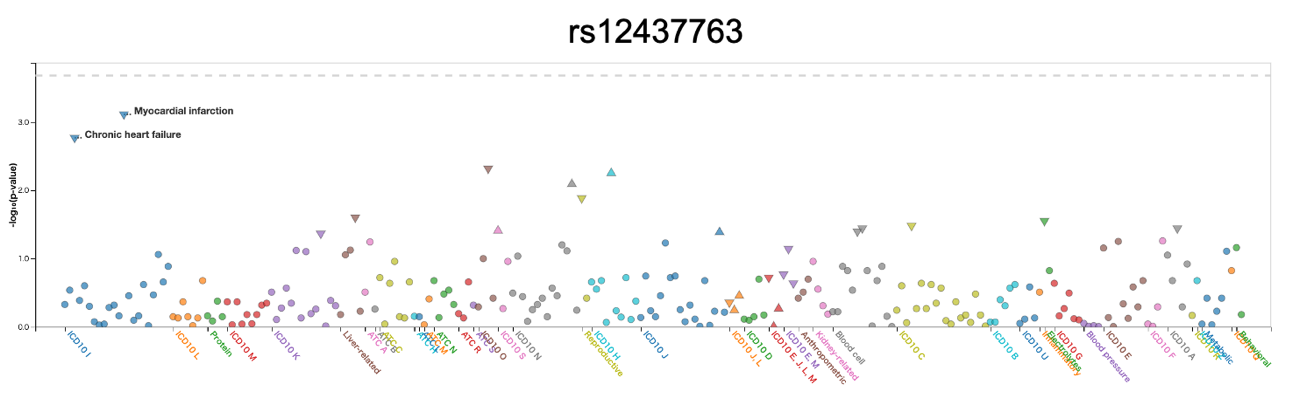


A

B

**Supplementary Figure 2 Manhattan plot of PheWAS for candidate SNPs.**

The plot displays the association between phenotypes and rs3176334 (A) and rs12437763 (B). The x-axis represents different traits categorized by ICD-10 codes and other categories, while the y-axis represents the negative logarithm of the *p*-value. Each point represents a phenotype and colors represent the trait categories. Triangles denote the traits with significant associations. The dashed grey line denotes an adjusted significance level.


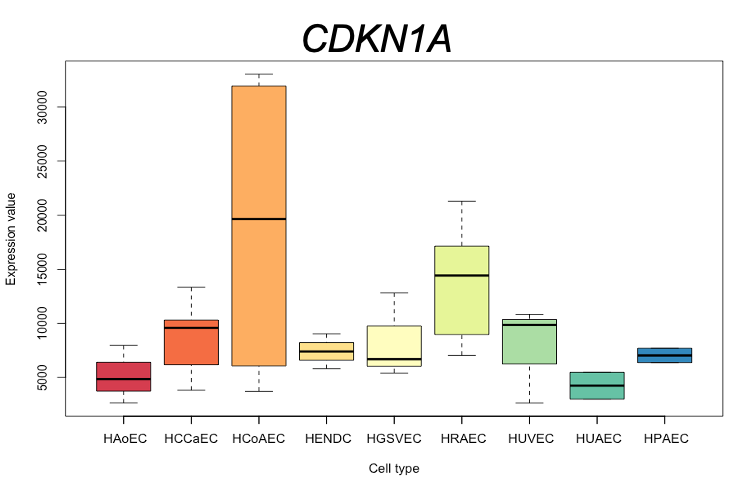

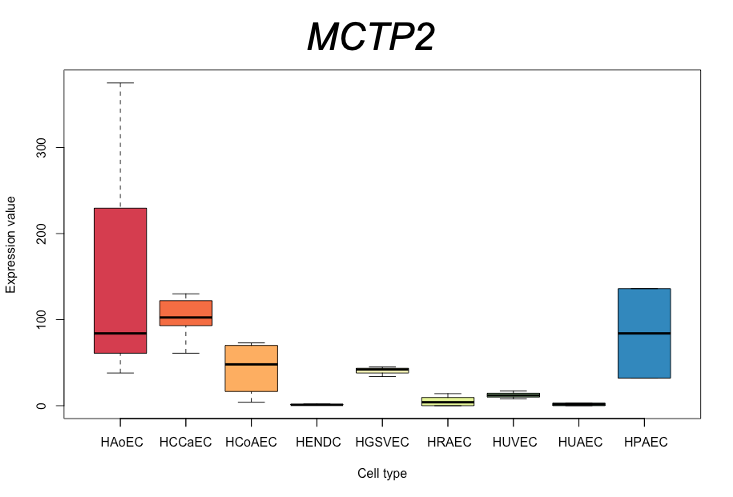


A

B


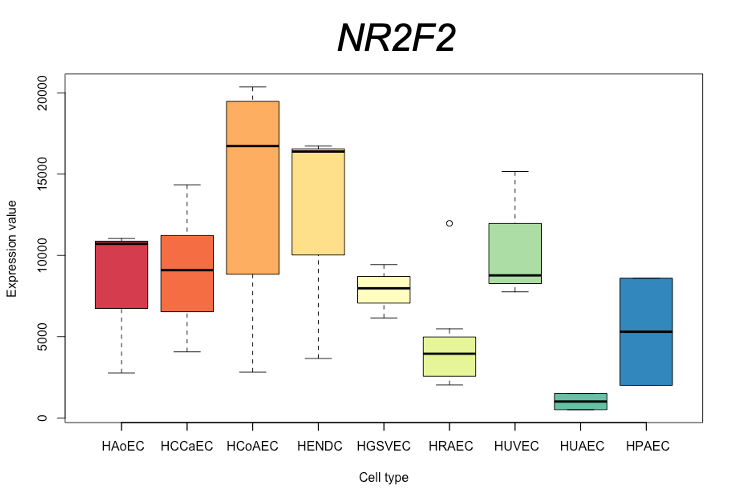


C

**Supplementary Figure 3 Expression levels of target genes of candidate SNPs across different endothelial cell types.**

Box plots show the expression levels of *CDNKN1A* (A), *MCTP2* (B) and *NR2F2* (C) in endothelial cells. Each columns represent the expression levels in human aortic endothelial cells (HAoECs), human common carotid artery endothelial cells (HCCaECs), human coronary artery endothelial cells (HCoAECs), human endocardial cells (HENDCs), human great saphenous vein endothelial cells (HGSVECs), human renal artery endothelial cells (HRAECs), human umbilical vein endothelial cells (HUVECs), human umbilical artery endothelial cells (HUAECs), and human pulmonary artery endothelial cells (HPAECs), respectively.


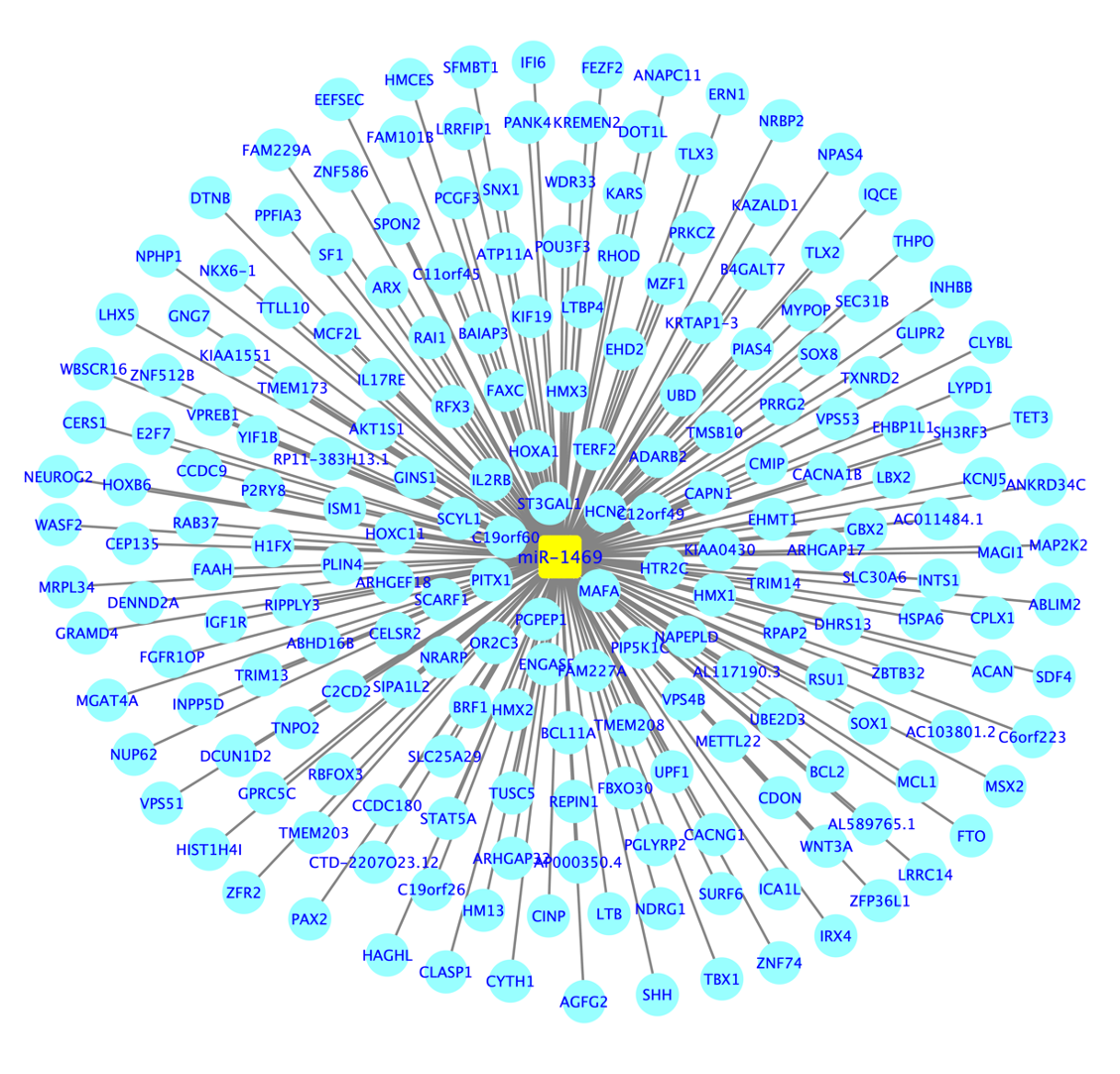


**Supplementary Figure 4 Interaction network of miR-1469 with target genes.**

The network diagram shows the interactions between miR-146 (highlighted in yellow at the center) and its predicted target genes (blue nodes). Each edge represents a regulatory interaction between miR-146 and its target gene.


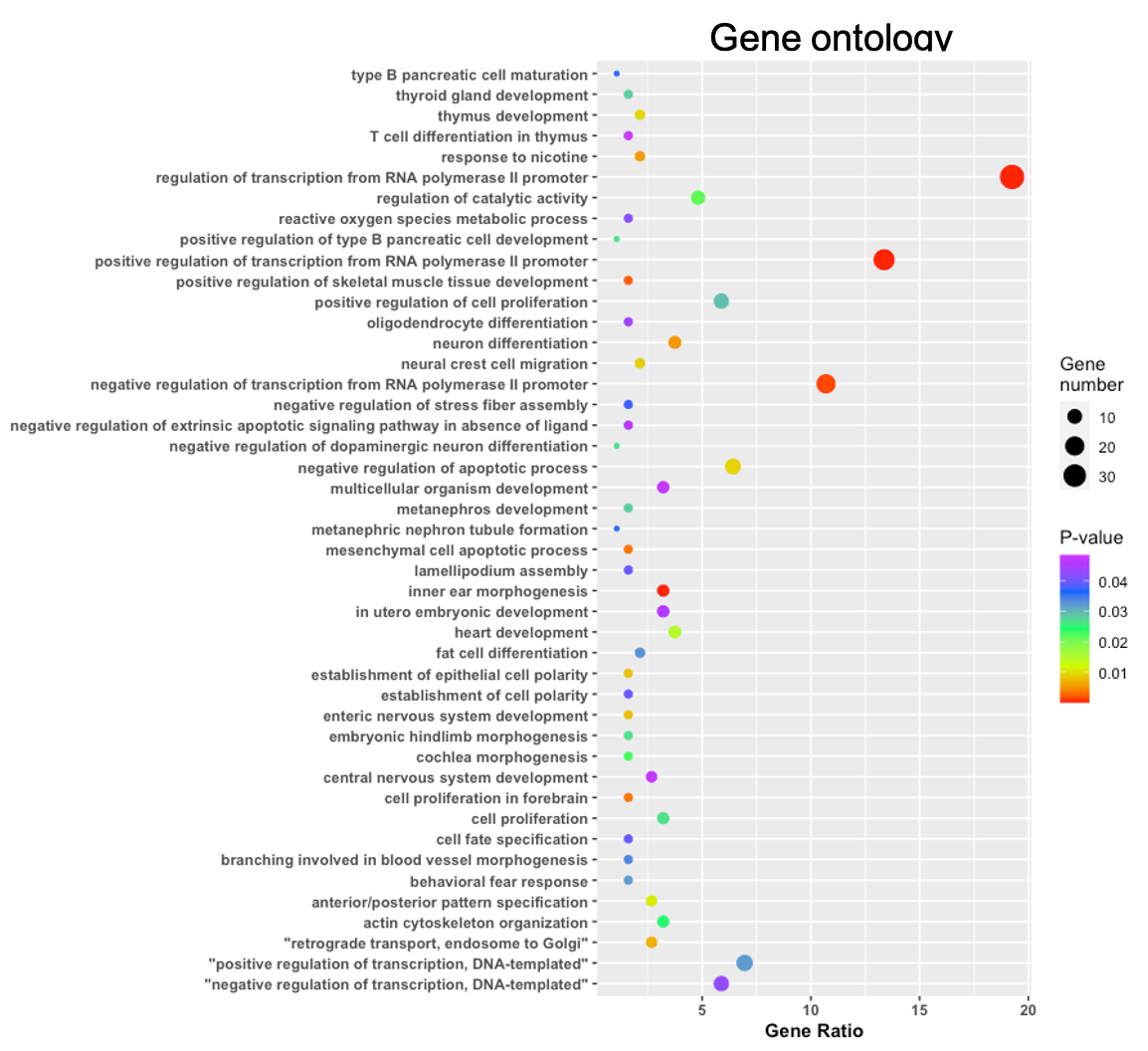


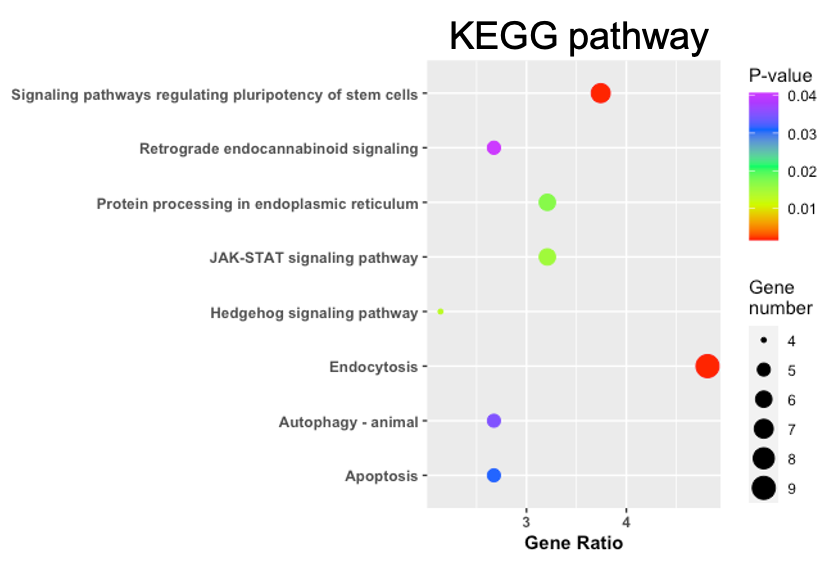


**Supplementary Figure 5 Gene ontology and KEGG pathway enrichment analysis of target genes interacted by miR-1469.**

Enriched GO terms (A) and KEGG pathway (B) of target genes interacted by miR-1469. The y-axis denotes GO terms (A) and pathways (B), whereas the x-axis denotes the gene ratio. The size of each circle represents the number of genes. The color scale represents the *p*-values.

A

B

*
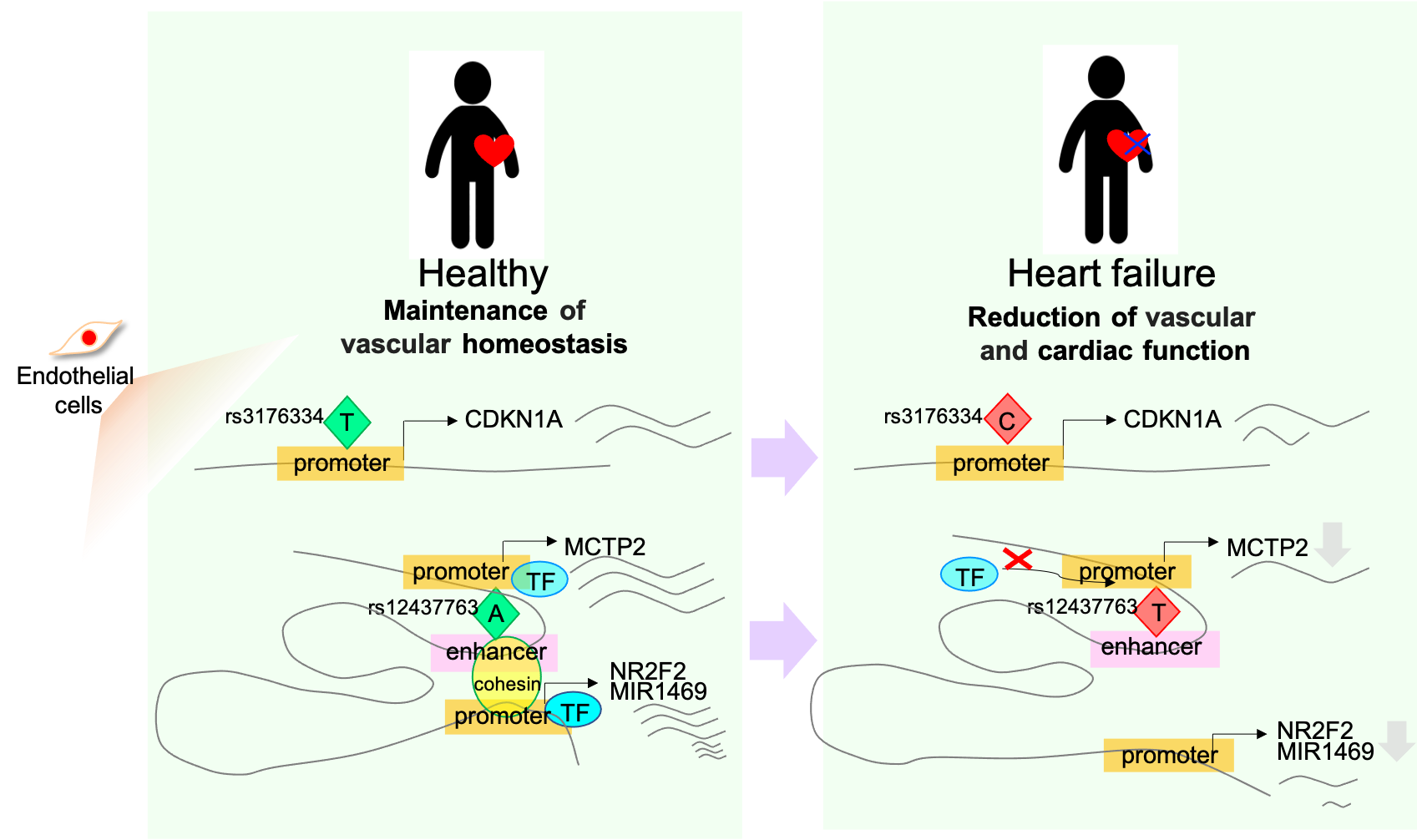
*

**Supplementary Figure 6 Scheme of the predicted molecular mechanism by which rs12437763 influences the risk of Japanese heart failure**

(A) The putative molecular mechanism associated with the reference alleles (rs3176334-T and rs12437763-A). rs3176334-T regulates the normal expression of *CDKN1A*, and the enhancer containing rs12437763-A forms chromatin loops that govern the stable expression of *NR2F2* and *MCTP2*.

(B) The putative molecular mechanism linked to the alternative alleles (rs3176334-C and rs12437763-T). rs3176334-C affects *CDKN1A* expression and splicing patterns, whereas rs12437763-T hinders chromatin loop formation, destabilizing the expression of *NR2F2* and *MCTP2*, identified as risk factors for heart disease.
